# COVID-19 Pneumonia Classification Based on NeuroWavelet Capsule Network

**DOI:** 10.3390/healthcare10030422

**Published:** 2022-02-23

**Authors:** Happy Nkanta Monday, Jianping Li, Grace Ugochi Nneji, Saifun Nahar, Md Altab Hossin, Jehoiada Jackson

**Affiliations:** 1 School of Computer Science and Engineering, University of Electronic Science and Technology of China, Chengdu 611731, China; mh.nkanta@std.uestc.edu.cn; 2 School of Information and Software Engineering, University of Electronic Science and Technology of China, Chengdu 611731, China; ugochinneji@std.uestc.edu.cn (G.U.N.); kofijackson@uestc.edu.cn (J.J.); 3 Department of Information System and Technology, University of Missouri St. Louis, St. Louis, MO 63121, USA; snnnm@umsl.edu; 4 School of Management and Economics, University of Electronic Science and Technology of China, Chengdu 611731, China; altabbd@uestc.edu.cn

**Keywords:** COVID-19, chest X-ray, convolutional neural network, wavelet, capsule network, pneumonia

## Abstract

Since it was first reported, coronavirus disease 2019, also known as COVID-19, has spread expeditiously around the globe. COVID-19 must be diagnosed as soon as possible in order to control the disease and provide proper care to patients. The chest X-ray (CXR) has been identified as a useful diagnostic tool, but the disease outbreak has put a lot of pressure on radiologists to read the scans, which could give rise to fatigue-related misdiagnosis. Automatic classification algorithms that are reliable can be extremely beneficial; however, they typically depend upon a large amount of COVID-19 data for training, which are troublesome to obtain in the nick of time. Therefore, we propose a novel method for the classification of COVID-19. Concretely, a novel neurowavelet capsule network is proposed for COVID-19 classification. To be more precise, first, we introduce a multi-resolution analysis of a discrete wavelet transform to filter noisy and inconsistent information from the CXR data in order to improve the feature extraction robustness of the network. Secondly, the discrete wavelet transform of the multi-resolution analysis also performs a sub-sampling operation in order to minimize the loss of spatial details, thereby enhancing the overall classification performance. We examined the proposed model on a public-sourced dataset of pneumonia-related illnesses, including COVID-19 confirmed cases and healthy CXR images. The proposed method achieves an accuracy of 99.6%, sensitivity of 99.2%, specificity of 99.1% and precision of 99.7%. Our approach achieves an up-to-date performance that is useful for COVID-19 screening according to the experimental results. This latest paradigm will contribute significantly in the battle against COVID-19 and other diseases.

## 1. Introduction

After its first report in December 2019, COVID-19 has rapidly spread worldwide. The disease is extremely infectious and, as of 3 October 2021, it had infected over 234 million people worldwide, resulting in over 4.7 million deaths [1,2]. According to [1], the golden rule for detecting COVID-19 is reverse transcriptase polymerase chain reaction (RT-PCR) tests or a nasopharyngeal swab. RT-PCR, on the other hand, has been found to have an inadequate sensitivity, with a high ratio of false negatives for successful early detection and successive treatment of possible patients in several studies [3,4,5].

Meanwhile, inhaling symptoms (primary pneumonia) are common with COVID-19 patients [6]. Computed tomography (CT)-based non-contrast thoracic is becoming a viable option for diagnosing COVID-19-confirmed patients with suspected complications. Quite a number of studies [4,5,6] identified diffuse ground-glass opacities, especially peripheral and bilateral ground-glass, including consolidative pulmonary opacities, as typical COVID-19 CT findings.

Furthermore, ref. [7] suggested a scheme named the modified enhanced super resolution generative adversarial network for improving the resolution of computed tomography images. In contrast to the common style of increasing the depth of network and complexity to boost imaging performance, the authors integrated a Siamese capsule network that extracts distinct features for COVID-19 identification. Furthermore, both [3,4] found that non-contrast CT had a higher RT-PCR sensitivity. However, due to the sudden outbreak of the COVID-19 pandemic, experts are now under immense pressure to read the massive number of CT exams. One thin-slice CT series of 300 slices will take a radiologist 5–15 min to diagnose. Aside from being time-consuming and tedious, the reading process can also be susceptible to errors and omissions due to the heavy workload. In addition, junior radiologists may have difficulty in detecting early signs of COVID-19 in CT scans. COVID-19 detection using CT scans is time-consuming and manual, and it necessitates the intervention of experts. CT scanning equipment are often troublesome to operate for COVID-19 patients since they must often be moved to the CT room. The equipment must be thoroughly cleaned after each use, and there is a higher risk of radiation exposure. CT has been successfully used as a supportive method for COVID-19 condition evaluation despite the fact that it is not approved as a basic diagnostic means [8].

Using deep learning models to automate COVID-19 diagnosis from CT images has shown promising results in several studies [8,9,10]. Both RT-PCR tests and CT scans are relatively expensive [11,12] and quite a number of nations are mandated to conduct limited testing for only risk-prone populations due to excessive demand. CXR imaging is a comparably low-cost method of detecting lung infections, and it can also be used to detect COVID-19 [13,14,15,16,17]. As a result, automated algorithms that can accurately classify COVID-19 from CXR exams are extremely useful in the fight against the pandemic.

Deep learning (DL)-based approaches have been utilized for a variety of problems in medical image processing in recent years, with excellent results [18,19,20], including the detection of pediatric pneumonia using CXR images [21]. It is important to mention that DL-based approaches are considered to be data-hungry. In most cases, collecting a large dataset to train DL-based models for an emerging disease, such as COVID-19, is difficult for a lot of healthcare centers, particularly at the early stage of the outbreak, because a large dataset collection is needed. A range of medical biomarkers and abnormalities have also been investigated as indicators of disease development in research, and there are some indications that imaging data could supplement these models [22,23,24].

Bertini et al. [25] suggested an autoencoder neural network with a strategy of data augmentation to automatically classify visual representations of the audio data of patients living with dementia according to the advancement stage of the pathology. The authors utilized 288 audio dataset from 96 individuals, with a total number of 48 healthy controls and 48 cognitively impaired patients. An ECG-based method of preprocessing outliers and classifying BG ranges was suggested by Enayati et al. [26], which combined density-based spatial clustering applications with noise and convolution neural networks (DBSCAN-CNN). With the suggested strategy, the non-invasive monitoring of three BG, such as low glucose, moderate glucose and high glucose, were achieved. Nneji et al. [27] suggested a scheme that combines a wavelet transform and generative adversarial network (GAN) CNN in order to enhance the low quality of radiograph images for COVID-19 identification. The study of attack detection and ECG-based biometric identification has utilized the DL algorithm combined with wavelet multi-resolution analysis extensively [28,29].

On the one side, the number of positive cases (for example, COVID-19) is very low. On the other hand, these medical centers are more likely to have a large archive of other pneumonia cases and non-pneumonia CXR data. As a result of the large number of parameters, CNNs can overfit on small dataset. As a result, the generalization efficiency is reciprocal to the size of the labeled data. A small dataset is the most difficult task in the medical imaging domain because of the restricted quantity and range of samples [30,31,32]. Medical image mining is a time-consuming and costly procedure that necessitates the involvement of radiologists and researchers [31].

Furthermore, due to the recent nature of the COVID-19 outbreak, adequate data (CXR) are difficult to come by. However, in AI-based COVID-19 screening systems from CXR imaging, there are two major problems: (1) loss of spatial details due to the down-sampling operation during high-level feature extraction and (2) the image quality is still a major concern, as this may vary among samples, which, most times, include noisy and irrelevant annotations. The consequent of this is that the AI-based system will learn inconsistent and noisy information from the data, thereby missing the distinct features that would have been extracted for optimal classification.

We suggest a novel solution to this problem in this paper in order to successfully train DL-based algorithms to examine COVID-19 on CXR scans, and specifically to differentiate COVID-19 CXR from healthy CXR and non-COVID-19 CXR. We proposed a neurowavelet capsule network for COVID-19 pneumonia classification. The contributions of this work include: (1) to improve the feature extraction robustness of the network by introducing a multi-resolution analysis of a discrete wavelet transform to filter noisy and inconsistent information from the CXR data, and (2) to minimize the loss of spatial details by introducing a discrete wavelet multi-resolution analysis to perform a down-sampling operation. From what we can tell, this paper is the first work that introduces a multi-resolution analysis and capsule network as a mutual learning for feature extraction from CXR images for fast and efficient COVID-19 classification.

The proposed method is an end-to-end framework of a multi-resolution analysis of a discrete wavelet transform and capsule network for COVID-19 classification that achieves a much higher diagnosis accuracy. The subsequent part of this study is coordinated as follows: Section 2 will survey related essays. Section 3 will give a detailed explanation of the methodology and dataset description. Descriptive information about the experiment results is presented in Section 4. Finally, Section 5 concludes this study.

## 2. Related Works

Previously, DL-based approaches have been utilized to reliably investigate a range of ailments from medical images, surmounting human weakness in many cases. In addition, DL has recently been used to detect COVID-19 in medical videos. A custom CNN residual architecture was proposed in [33] to identify healthy, COVID-19 or pneumonia images. The algorithm was validated on a publicly accessible dataset [34]. The model achieved an 80% sensitivity and 99.5% specificity on just 10 COVID-19 data instances. To minimize the number of false negatives, future models should increase the sensitivity, according to this research. A ResNet-50-based Bayesian convolutional neural network (BCNN) was proposed in [35], with a strategy of dropping the weights, as inspired in [36]. The research dataset included 14 COVID-19 images in total, in which, two of the COVID-19 cases were incorrectly categorized in BCNNs and CNNs with a variety of weights dropped. Their best model for COVID-19 diagnosis had an 86% sensitivity and 99% specificity. Different saliency maps were applied to decrease the black-box behavior of deep learning. However, the maps only spot some incorrect areas, which were not examined in the study.

The study presumed that, by measuring the contingency within predictions, the model effectiveness could be enhanced. To diagnose COVID-19, three separate pre-trained deep learning networks were suggested by the researchers in [37] using 50 healthy and 50 COVID-19 instances. They achieved satisfactory results, where Inception V3 and ResNet-50 gave an impressive result whereas Inception-ResNet V2 incorrectly classified one healthy image as COVID-19 in a testing collection of ten images from each class [38,39,40,41]. They recommended the utilization of transfer learning for constructing deep learning models for COVID-19 screening. The authors in [42] proposed an 18-layer residual network that consisted of a sigmoid activation function immediately after the fully connected layers for COVID-19 classification. At the end of the CNN, a separate anomaly detection mechanism was introduced. In addition, the specificity and sensitivity were noted at specific thresholds in a system of two-fold cross-validation using 100 scans from 70 victims, achieving a 72% sensitivity and 98% specificity, as well as a 96% sensitivity, and 70% specificity.

In conclusion, these models tend to work admirably; however, due to the possibility of missing a COVID-19 diagnosis, increasing the model sensitivity is a top priority. More models have been developed to diagnose COVID-19 using CT and CXR scans [43]. At first, the authors trained the algorithm to extract COVID-19 regions from professional annotated CT slices with 16 suspected pneumonia scans and 11 COVID-19 exams. They came to the conclusion that their approach obtained a comparable outcome to professional radiologists, achieving a 100% sensitivity in the patient category. A similar algorithm was proposed in [44] with a slightly different network for COVID-19 segmentation and quantification, obtaining a dice record of 91.6% using 300 COVID-19 exams. The authors suggested that their approach could be used to monitor the disease’s progression. COVID-19 was segmented and quantified using a combination of commercial software and deep learning in [45], achieving 99.6% AUC. However, a patch-based integrated support vector machine method was suggested in [46], with a performance of 100% specificity and 93% sensitivity.

A weakly supervised approach was proposed in [47]. This approach produced segmentation masks automatically. For classification, the mask and CT image are introduced into a 3D CNN. This procedure obtained 96% AUC. In brief, most essays that have utilized X-ray imaging rely on quite a large amount of COVID-19 images from various sources with no standardized protocols. The algorithms they implemented are only simple extensions of previously existing ones. The overall COVID-19 image-based models perform well; however, some models utilize as few as 10 images, whereas others rely on external validation due to the issues of data availability according to [48]. The goal of our research is to develop a research methodology that will leverage the technique of the multi-resolution analysis and capsule network to achieve up-to-date results.

## 3. Materials and Methods

### 3.1. Wavelet

Wavelets are a type of function used to scale and localize functions. The wavelet transform splits the input image into multiple frequency constituents, then analyzes each one with a resolution appropriate for its scale. The wavelet transform works by extending and converting an input image in the time domain using a wavelet basis, then decomposing it into a sequence of sub-band components with varying image resolution, frequency properties and directional features. Low frequency constituents are kept in the wavelet transform to achieve dimensionality reduction, whereas high frequency constituents are discarded as much as possible. The term “wavelet” refers to a “small wave” function, generally indicated as ψ(·), defined over the main axis (−∞,∞). It must fulfill the basic three properties to be classified as a wavelet, as presented in Equations (1)–(4).

The integral of ψ(·) is zero, as presented in Equation (1).

(1) $$\int_{-\infty }^{\infty }\psi \left(u\right)du=0$$

The integral of the square of ψ(·) is unity, as presented in Equation (2).

(2) $$\int_{-\infty }^{\infty }\psi^{2}\left(u\right)du=1$$

Equation (3) explicitly expresses the admissibility condition.

(3) $$C_{\psi }=\int_{0}^{\infty }\frac{{\left|\psi \left(f\right)\right|}^{2}}{f}\;df$$

By converting and stretching this mother wavelet, as shown in Equation (3), a two fold-indexed family of wavelets can be formed.

(4) $$\psi_{\lambda ,t}\left(u\right)=\frac{1}{\sqrt{\lambda }}\psi \left(\frac{u-t}{\lambda }\right)$$

where λ>0 and *t* is 1, the normalization on the right hand side of Equation (4) is chosen such that $\left|\right|\psi_{\lambda ,t}\left|\right|=\left||\psi |\right|$ for all λ and *t* and $\frac{1}{\sqrt{\lambda }}$ is the normalizing term.

### 3.2. Multi-Resolution Analysis (MRA)

Multi-resolution analysis (MRA) is the foundation of the wavelet principle, which separates an image into wavelets (wave-like functions) that are scaled and time-shifted replicas of the original or mother wavelet. Scaling and wavelet functions are used to implement low and high pass filters, respectively. As a result, after passing through the low and high pass filters, the image is sub-sampled to discriminate low and high frequencies. The relationship between the original image and the decomposition components f(t) is expressed in Equation (5).

(5) $$f\left(t\right)=CA_{4}+CD_{4}+CD_{3}+CD_{2}+CD_{1}$$

where f(t) is the original image, $CA_{4}$ is the approximate component of the fourth level decomposition and $CD_{4}$, $CD_{3}$, $CD_{2}$ and $CD_{1}$ are the detail components for the fourth, third, second and first level decompositions, respectively. Multi-resolutional analysis uses the discrete wavelet transform (DWT) approach to describe a time-varying signal in terms of frequency elements. The original image is split into several additional images of various resolutions (scale). The image f(t) is disintegrated into scaling and wavelet functions, which can be expressed mathematically in Equation (6).

(6) $$f\left(t\right)=\sum_{k}A_{j}\left(k\right)⌀\left(t-k\right)+\sum_{k}\sum_{j=0}^{1}D_{j}\left(k\right)2^{\left(j⁄2\right)}\psi \left(2^{j}t-k\right)$$

**Figure 1:**
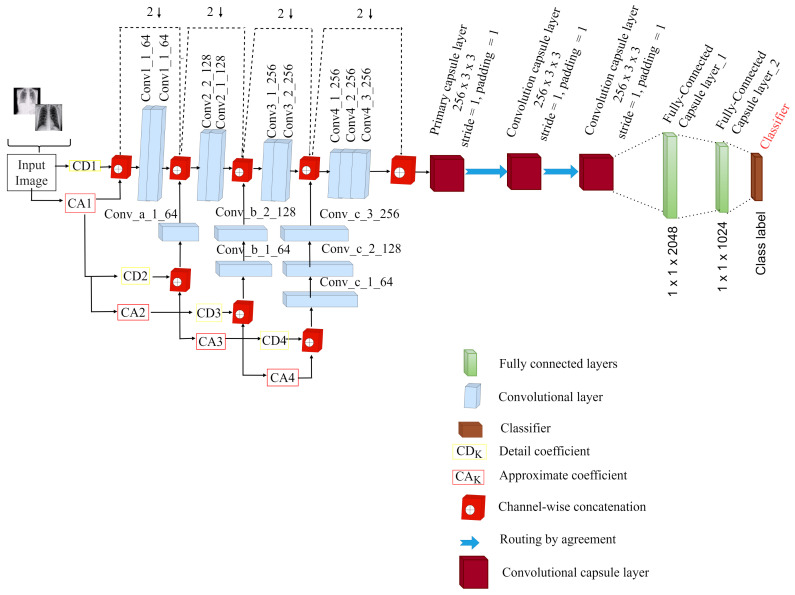
Our proposed neurowavelet capsule network for COVID-19 classification (NW-CapsNet).

As shown in Figure 1, the wavelet function $\psi (2^{j}t-k)$ generates the high frequency constituents (detailed) of the disintegrated image, whereas the scaling function ⌀(t−k) generates the low frequency constituents (approximate). The frequency constituents of detail and approximates are extracted via a filter bank comprising high pass and low pass filters. For each stage of decomposition, the wavelet is scaled by a factor of two. To obtain more information about the input image, the low frequency constituent is decomposed again. The beginning section of the right side is a projection of f(t) in the scaling space, with coefficients $A_{j}\left(k\right)$ representing image f(t)’s discrete smoothing approximations, and the other section is a projection of f(t) in the wavelet space, with coefficients $D_{j}\left(k\right)$ representing image f(t)’s discrete informative features of the image, which are the wavelet transform coefficients. In image processing applications, wavelet multi resolution analysis is widely used and efficient. This paper combines the technique of multi-resolution analysis of discrete wavelet transform and capsule network for COVID-19 classification task, as illustrated in Figure 1. For the image input, the discrete wavelet transform (DWT) is presented numerically in Equations (7) and (8).

(7) $$W_{⌀}\left(j_{0},z_{1},z_{2}\right)=\frac{1}{\sqrt{M\times N}}\sum_{x=1}^{M}\sum_{y=1}^{N}I(x,y){⌀}_{j_{0},z_{1},z_{2}}\;\left(x,y\right)$$

(8) $$W_{\psi }\left(j_{0},z_{1},z_{2}\right)=\frac{1}{\sqrt{M\times N}}\sum_{x=1}^{M}\sum_{y=1}^{N}I(x,y)\psi_{j_{0},z_{1},z_{2}}^{i}\;\left(x,y\right)$$

where M×N indicates image dimension, I(x,y) is the pixel intensity at position (x,y), *⌀* and ψ are the scaling and wavelet functions and i=(H,V,D) is the wavelet function’s path index. Wavelet function generates 4 sub-bands for one image at separate level ψ: smooth version (LL), vertical borders (LH), horizontal borders (HL) and diagonal borders (HH) of the image.

### 3.3. Multi-Resolution Analysis of Discrete Wavelet CNN

As shown in Figure 1, the proposed Neurowavelet architecture is a discrete wavelet multi-resolution integrated convolutional neural network with 9 convolutional layers grouped in blocks and a four-level decomposition of the multi-resolution wavelet transform. Each block of the convolutional layers receives input from each level of the decomposition. The detail coefficient obtained from the wavelet decomposition process is fed as input to the proposed network.

More precisely, first, we introduced multi-resolution analysis of discrete wavelet transform to filter noisy and inconsistent information from the CXR data in order to improve the feature extraction robustness of the network. Secondly, the discrete wavelet transform of the multi-resolution analysis also performed sub-sampling operation in order to minimize the loss of spatial details, thereby enhancing the overall classification performance. We used wavelet multi-resolution analysis technique to perform sub-sampling operation similar to conventional pooling layers by decomposing the input image feature into high- and low-frequency components of sub-bands with a scaling factor of 2. The size of the input data was 224×224×3. In the first level of decomposition, the input image was decomposed to generate low- and high-frequency image components, which reduced the input data size of 224×224×3 to a feature map size of 112×112×12, which was then concatenated to the first block of convolutional layers with 3×3 filter size and 64 kernel size. In the second level of decomposition, the low-frequency image component previously generated was decomposed and reduced to a feature map size of 56×56×12, which was then concatenated to the second block of convolutional layers with 3×3 filter size and 128 kernel size.

It is important to mention that, before the high-component was fed to the second convolutional block, it was concatenated channel-wise through 1×1 convolutional layer of 64 kernel size for the purpose of achieving dimensionality match with the output of the first convolutional block. In the third level of decomposition, the low-frequency image component previously generated was decomposed to generate low- and high-frequency image components, which were reduced to the feature map of 28×28×12 and then concatenated channel-wise to the third block of convolutional layers with 3 × 3 filter size and 256 kernel size via two 1×1 convolutional layers of 64 and 128 kernel sizes for the purpose of achieving dimensionality match with the output of the second convolutional block. Finally, in the fourth level of decomposition, the low-frequency image component previously generated was decomposed to generate low- and high-frequency image components, which were reduced to the feature map of 14×14×12 and then concatenated channel-wise to the fourth block of convolutional layers with 3×3 filter size and 256 kernel size via three 1×1 convolutional layers of 64, 128 and 256 kernel sizes for the purpose of achieving dimensionality match with the output of the second convolutional block. It is worth noting that the images were down-sampled by a factor of two throughout the wavelet decomposition process.

### 3.4. Proposed NeuroWavelet Capsule Network

In recent works, classical CNN has been used to obtain fantastic results in feature extraction for classification tasks. Traditional CNNs use scalar neurons to express the probabilities of specific features being present, which severely restricts their performance. More specifically, the pooling layer’s major goal is to achieve down-sampling and to minimize the dimension of the features, although this may result in a loss of spatial details, large rotation and directional route. This is one of the causes for CNN’s poor classification performance. In order to properly classify COVID-19, we presented a novel neurowavelet capsule network (NW-CapsNet). The authors of [49] are the first to suggest a capsule network as a solution to classification problems. Unlike standard CNNs, a capsule network consists of entity-inclined vectorial capsules. A capsule can be viewed as a vectorial cluster of neurons [50]. The initialization parameters of a capsule represent a certain type of entity, and the length of the capsule indicates the likelihood of that entity’s existence. Capsule networks are more powerful and reliable than standard CNNs at retrieving intrinsic and distinguishing features of entities [49,50,51].

To obtain promising COVID-19 diagnosis performance, we modified the existing capsule network by replacing the convolutional layers before the primary capsule layer with multi-resolution analysis discrete wavelet CNN as a feature extractor to create a multi-layer deep convolutional capsule network. Figure 1 shows a detailed depiction of our proposed neurowavelet capsule network (NW-CapsNet). The first few convolutional layers capture low-level information, such as curves, color, edges and texture, due to the discriminative features of the CXR scans; however the high-level features are captured as the convolutional layers grow deeper. The spatial details were transformed into primary capsule (PrimaryCaps) in the form of capsules immediately after the feature extraction stage. Routing by agreement was utilized to learn the spatial information in the form of a transformation matrix. The routing coefficient controls the connection strength of the digit capsule. The length of the feature vector which is compressed to 1 using a non-linear function, represents the probability that the capsule instance exists. The feature representations obtained after the feature extraction step is directly fed to the primary capsule. The numerical expression in Equation (9) shows that the total input in the convolutional capsule layers is the weighted sum of all predictions obtained from the capsules within the convolutional kernel.

(9) $$C_{j}=\sum_{i}a_{ij}\;\cdot \;U_{j/i}$$

where $C_{j}$ represents the sum total input to capsule *j*, and the indicator that depicts the degree of which capsule *i* activates capsule *j* is called the coupling coefficient $a_{ij}$. The prediction $U_{j/i}$ from capsule *i* to capsule *j* is expressed in Equation (10).

(10) $$U_{j/i}=W_{ij}\;\cdot \;U_{i}$$

$U_{i}$ denotes the capsule *i*’s output and $W_{ij}$ represents the weight network on the edge linking capsules *i* and *j*. A robust routing method [51] decided the coefficient between capsule *i* and other linked capsules in the above layer, summing to 1.

The robust routing approach, known as routing by agreement, considers both the length of a capsule and its instantiation parameters when igniting another capsule. Traditional CNN models, on the other hand, merely evaluate the probability. As a result, capsule networks are more capable of abstracting inherent image features and are more dependable. It is worth mentioning that the length of the capsule is used to estimate the possibility of an entity’s presence. In order to ensure perfect probability estimation, a non-linear “squashing” function is used as the activation function for the convolutional capsule layers, where capsules with short vectors have low probability and long vectors have high probability while maintaining a constant orientation. The numerical expression for the squashing function is given in Equation (11).

(11) $$U_{j}=\frac{\;||C_{j}{\left|\right|}^{2}}{1+\left|\right|C_{j}{\left|\right|}^{2}}\;\cdot \;\frac{C_{j}}{\left|\right|C_{j}\left|\right|}$$

The original capsule network did not utilize max pooling, yet achieved commendable performance, but incurred higher computational cost. One important goal of this paper is the implementation of discrete wavelet multi-resolution analysis prior to the capsule layers as a strategy to perform down-sampling operation in order to avoid the loss of spatial details, which is a common problem in traditional CNN, while maintaining the integrity of high-level features, as well as reducing the dimensionality of the network size.

To this effect, the size of the network and number of capsule were reduced, leaving only a few important capsules. In the three fully connected layers, a high-level entity abstraction with a global view was built. The squashing function was used to normalize capsule outputs, as well as dynamic routing between two connected capsule layers.

We used softmax in the last fully connected capsule layer because our class label was greater than two. To successfully train the capsule network for classification tasks, we used the margin loss [51] as the objective function to guide the error back-propagation process. The margin loss Lk for class *k* is expressed mathematically in Equation (12).

(12) $$L_{k}=T_{k}\;\cdot \;max{\left(0,\;\;m^{+}-U_{k}\right)}^{2}+\eta \left(1-T_{k}\right)\;\cdot \;max{\left(0,U_{k}-m^{-}\right)}^{2}$$

where $U_{k}$ is the capsule’s output in the softmax layer. $T_{k}$ is set to 1 if and only if a training sample is an instance of class *k*; otherwise, $T_{k}=0$. m+ and m− represent the lower and upper bounds, respectively, for the likelihood of training data becoming an instance of class *k* and the probability of a training data not being an instance of class *k*. They were set to m+ = 0.9 and m− = 0.1. The weight regularizer factor *n* was set to 0.5 by default. The sum of the losses of all class-oriented capsules on all training data is the overall loss of the capsule network.

### 3.5. Dataset

**Figure 2:**
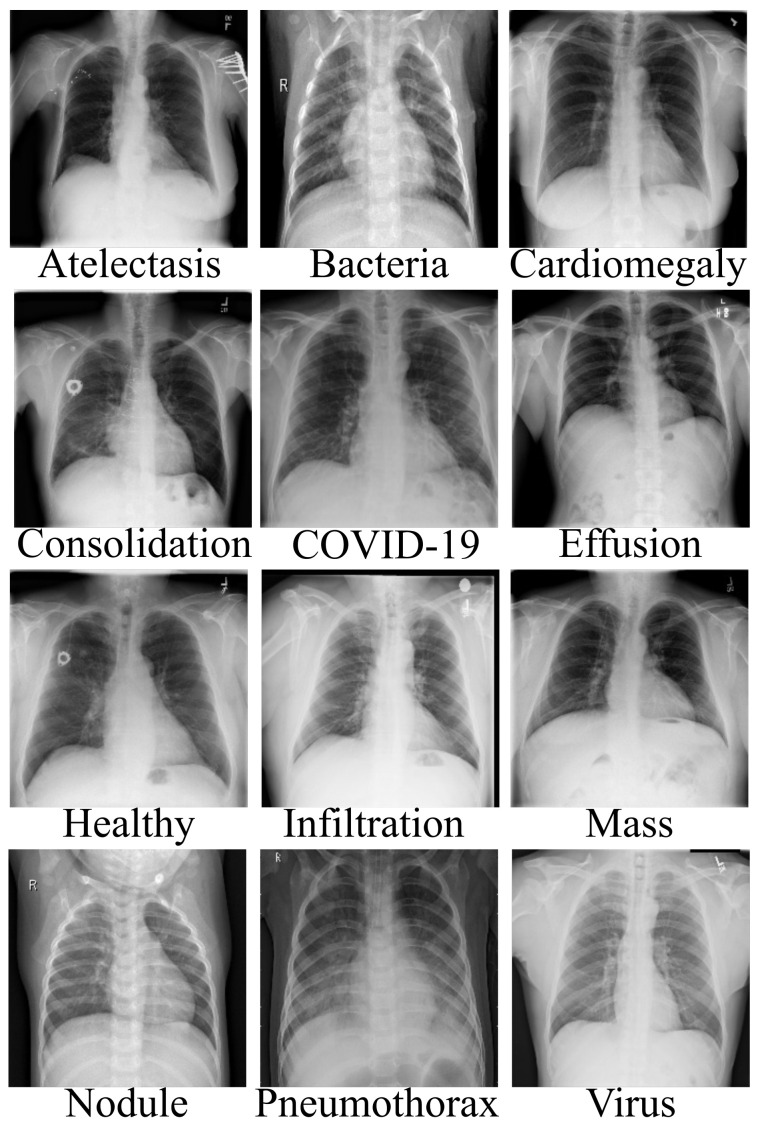
Data description of different pneumonia diseases, including COVID-19.

We used three separate open sources to acquire chest X-ray data of different pneumonia-related illnesses for this investigation [52,53,54]. As described in Table 1, we acquired 3616 scans of COVID-19 CXR from the COVID-19 radiography database [52]. We acquired 3029 scans of bacterial pneumonia, 8851 scans of healthy patients and 2983 scans of viral pneumonia from the Kaggle database of Radiological Society of North America (RSNA) [53]. In addition, we acquired 74,999 scans of other pneumonia-related illnesses from National Institute of Health (NIH) [54], as described in Table 1, for the purpose of validating our proposed architecture for multiple classification problems. As illustrated, there are approximately 94,627 CXR scans, including COVID-19 and 10 other pneumonia-related illnesses, as well as healthy instances. Since the number of each category of data class varies, as a result, we selected 2980 scans of CXR from each category, which sum up to 35,760 CXR images. In addition, since the amount of CXR associated with each class is balanced, the dataset was partitioned into three sets of 60%, 20% and 20% for training, validation and test, respectively. Figure 2 gives a visual representation of the dataset distribution.

## 4. Results

### 4.1. Experimental Setup and Details

To examine the performance of our proposed algorithm in screening COVID-19, we collected a dataset of chest X-ray scans from three open sources. We conducted a two-stage experiment to further validate the efficacy of our proposed model, with the first stage considering the proposed model with discrete wavelet multi-resolution analysis (MRA) and the second stage considering the proposed model with max-pooling. On the same dataset, we ran 11 well-known ImageNet pre-trained models and 4 cutting-edge COVID-19 methods for fair comparison.

In Table 2, we conducted two-stage experiments for the purpose of evaluating the influence of down-sampling operation on the classification performance of NW-CapsNet. The first experiment considered NW-CapsNet with max-pooling and the second experiment considered NW-CapsNet with discrete wavelet multi-resolution analysis (MRA). The result shows that our proposed NW-CapsNet with MRA is effective and achieves high diagnosis performance. The result in Table 2 shows that discrete wavelet multi-resolution analysis can improve the performance of capsule network, as well as being able to reduce the computational cost. It is important to mention that the training time of the proposed model with multi-resolution analysis is quite reduced compared to the model with max-pooling, as presented in Table 2. The proposed NW-CapsNet (MRA) achieved 99.6% accuracy with a training time of 23.2 min, which is quite commendable. It is evident in Table 3 that the computational cost of the proposed model is quite low when compared to the training time of the selected pre-trained models.

The proposed model is implemented on NVIDIA GTX1080 with a Keras framework. With a batch size of 16, we used a learning rate of $10^{-4}$. With the Adam optimizer, the model is trained for 30 epochs. To minimize over-fitting, we used a 50% dropout for regularization and batch normalization (BN). As previously stated, the dataset is divided into three sections: training, validation and testing. The model is equally verified for performance during the training phase. To acquire the final performance, the proposed model is evaluated using the test dataset. The accuracy (ACC), sensitivity (SEN), ROC and specificity (SPE) are the assessment criteria used to evaluate the performance of our proposed method. All indicators point out that our suggested model outperforms previous methods, including deep learning models, with promising results.

(13) $$Accuracy=\frac{TP+TN}{TP+TN+FP+FN}$$

(14) $$Sensitivity=\frac{TP}{TP+FN}$$

(15) $$Specificity=\frac{TN}{TN+FP}$$

where TP, FP and FN indicate the outcomes of true positive, false positive and false negative, respectively.

### 4.2. Discussion

Channel-wise concatenation was adopted to integrate varying scales of the input image into the convolutional neural networks to improve the performance of our proposed network (NW-CapsNet). The main goal of introducing the wavelet multi-resolution analysis (WMRA) is to obtain a full-spectral analysis by providing a varied depiction of the input images at different scales. The discrete wavelet multi-resolution analysis can interpret the input images at various scales. It is well-known that CNNs process images mainly in the spatial domain and only partially in the spectral domain, whereas a discrete wavelet multi-resolution analysis gives permission for the full-spectral processing of the input image.

By incorporating the discrete wavelet multi-resolution analysis into the convolutional neural network, it improves the network’s ability to extract the magnitude of the frequency data that are not obtained in the convolutional layers. Wavelets also extract the appropriate multi-resolution spectral information from the input data at different phases. Similar to how pooling works, a multi-resolution analysis of the data provides the input in several scales.

A wavelet transform works in such a way that every sub-sampling stage can be seen as a distinct pooling process. This triggered us to employ the discrete wavelet transform as a clear substitute for the pooling layers in the proposed framework utilized in this study, and also to extract information from the input data and pass it into the convolutional layers.

**Figure 3:**
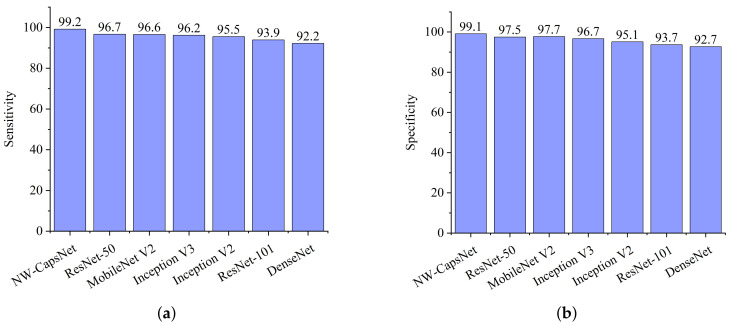
Comparison report of our model and selected pre-trained models. (**a**) Sensitivity result of the pre-trained models, including our proposed NW-CapsNet model. (**b**) Specificity result showing that our proposed model outperforms the selected pre-trained models.

The experiment revealed that our proposed NW-CapsNet architecture outperforms state-of-the-art COVID-19 models and a few pre-trained deep learning models on ImageNet. All implementations are based on their source code while using the same CXR dataset for fairness. From the comparative report of our experimental investigation, as presented in Figure 3a, DenseNet achieves the lowest sensitivity score of 92.2% and the lowest specificity score of 92.7%, as depicted in Figure 3b. From the results presented, our proposed model outweighs all the pre-trained models, with a high sensitivity score of 99.2% and a 99.1% specificity score.

In addition, the classification performance of the state-of-the-art COVID-19 models is reported in comparison to our proposed model, as presented in Table 4 whereas Table 5 shows the classification performance of the proposed model in comparison with the selected COVID-19 state-of-the-art methods using the same dataset. Our model performs better than the selected state-of-the-art models, achieving a high accuracy of 99.6%, followed by COVID-Net with 97.9% accuracy.

**Figure 4:**
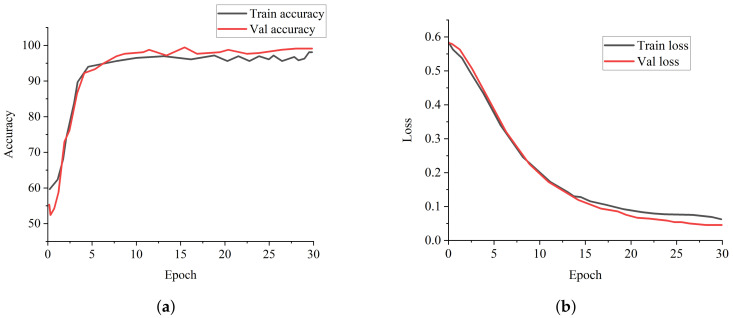
Training and validation curves for the proposed model. (**a**) Accuracy curve showing the performance of our proposed NW-CapsNet model. (**b**) The loss curve of our proposed NW-CapsNet model showing the stability of our model.

**Figure 5:**
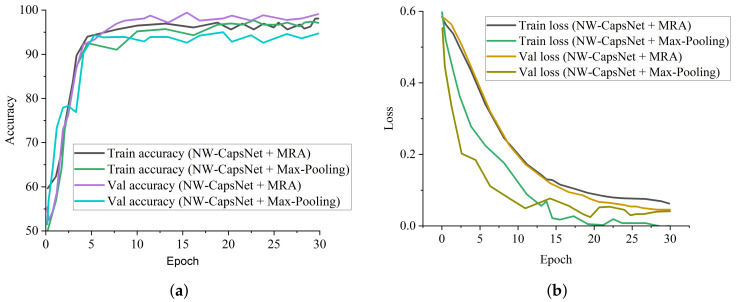
Training and validation curves for the proposed model with different down-sampling operations. (**a**) Accuracy curves showing the influence of the choice of down-sampling operation on the overall performance of the model. (**b**) The loss curves for our proposed model showing the influence of the choice of down-sampling operation.

ResNet-50 and COVID-Net perform well among the state-of-the-art COVID-19 models and pre-trained models, but our proposed model achieves the best results across all of the metrics. Figure 4a is the training and validation accuracy curves of our proposed model, depicting that our proposed NW-CapsNet converges steadily and fast. We further show that our proposed model converges smoothly and steadily with a moderate loss decrease, as shown in Figure 4b. The result of the experiment conducted with different choices of down-sampling operations shows that discrete wavelet multi-resolution analysis as a down-sampling operation enhances the performance of the model compared to the traditional max-pooling. We added a pooling layer in the capsule network to reduce the dimension of the capsule before passing it to the fully connected capsule layer. Figure 5a shows the training and validation accuracy curves of the two models with different choices of pooling operations, and Figure 5b shows the curves for both the training and validation loss. From all indications, the model with the discrete wavelet multi-resolution analysis converges smoothly, with a steady progression in accuracy and steady reduction in loss.

**Figure 6:**
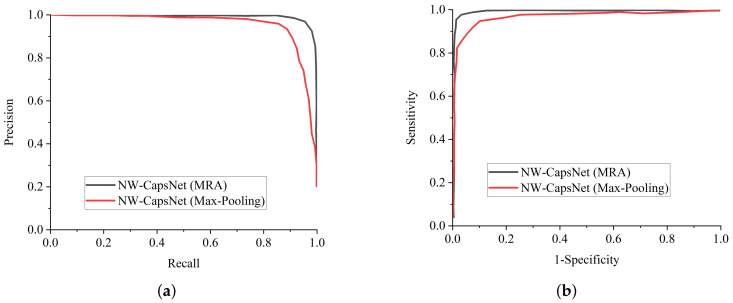
Effect of down-sampling operation on the performance of the proposed model. (**a**) Precision–recall curves of our two stage experiments showing the influence of down-sampling operation on the model performance. (**b**) ROC curve of our two stage experiments showing the influence of down-sampling operation on the model performance.

**Figure 7:**
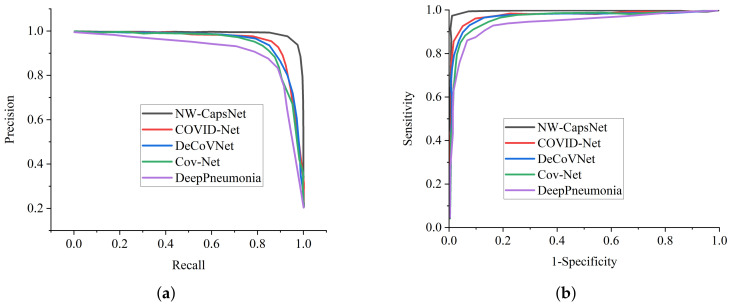
Performance comparison with state-of-the-art models. (**a**) Precision–recall curves of our proposed NW-CapsNet model in comparison to some state-of-the-art COVID-19 methods. (**b**) ROC curve of our proposed NW-CapsNet model in comparison to some state-of-the-art COVID-19 methods.

To further validate the efficacy of our proposed NW-CapsNet model, we adopted precision–recall and ROC. For diagnosing sensitive conditions, such as COVID-19, it is important to adopt ROC as a method to measure the overall accuracy, and the precision–recall curve to measure the mean average precision of our model. Figure 6a shows the precision–recall curve for the two stage experiment conducted with different choices of pooling operations, and the ROC curve is presented in Figure 6b. We went a step further and compared our proposed model with some selected state-of-the-art COVID-19 methods in terms of precision–recall and ROC, as presented in Figure 7a,b.

**Figure 8:**
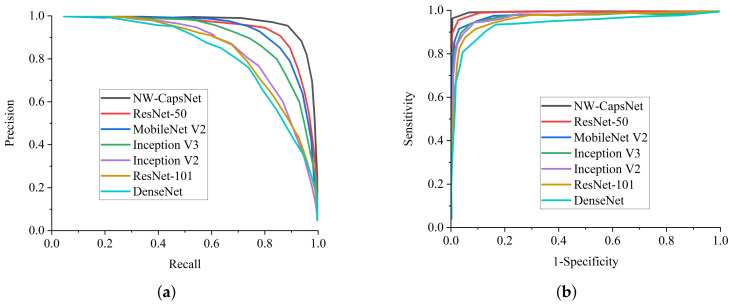
Performance comparison with state-of-the-art models. (**a**) Precision–recall curves of our proposed NW-CapsNet model in comparison to some selected pre-trained deep learning models. (**b**) ROC curve of our proposed NW-CapsNet model in comparison to some selected pre-trained deep learning models.

Finally, we compared our proposed model with some pre-trained deep learning methods in terms of precision–recall and ROC, as seen in Figure 8a,b. It is worth mentioning that all of the models are trained on the same dataset for fair comparison. We only modified the last layer of the models to correspond to the number of class labels in our dataset. From all indications, our proposed NW-capsNet outperforms the other models in terms of precision–recall and ROC. The precision–recall graphs show that the curves of our proposed model is the closest to the upper right corner of the graph with the largest area, and therefore has a higher precision associated with a higher sensitivity. Similarly, the ROC graphs indicate that the curves of our proposed model are the closest to the upper left corner of the graph, with the largest area under the curve, and therefore has a higher sensitivity associated with a higher specificity. More importantly, as mentioned above, the stated result in terms of the receiver operating characteristic (ROC) and precision–recall can assist expert radiologists in striking a balance between accuracy and precision.

We evaluated many literature connected to COVID-19 diagnosis using artificial intelligence and gave some comparisons. As seen in Table 4, some literature presented only a few performance metrics to back up their assertions. More specifically, as compared to other state-of-the-art COVID-19 approaches listed in the literature, our suggested model obtains superior performance, with more indicators reported.

### 4.3. Comparative Study

The result of our proposed model is compared to prior COVID-19 screening approaches. Many studies have been conducted to diagnose COVID-19 from CT and CXR images. The result of the proposed neurowavelet capsule network is compared to existing studies. A U-Net-based model was suggested by Chen et al. [8] to extract features of high resolution from CT, achieving a 95.2% accuracy, 100% sensitivity and 93.6% specificity. However, our model outperforms Chen et al. [8] in terms of the accuracy and specificity, with a margin of 4.59% and 6.26%, respectively. Wang et al. [31] used the CNN technique to detect COVID-19 and obtained a 93.3% accuracy, 87.6% sensitivity and 95.5% specificity. Our model obtained much higher results than Wang et al. [31] in terms of accuracy, sensitivity and specificity, with a margin of 5.89%, 12.19% and 4.36%, respectively. Classifying COVID-19 by utilizing a random forest technique was suggested by Shi et al. [55], achieving an 87.9% accuracy, 83.3% sensitivity and 90.7% specificity. A system of a logistic regression approach was adopted by Jin et al. [57] to detect COVID-19. The authors claimed that their model achieved a 97.4% sensitivity and 92.2% specificity.

Li et al. [45] applied the method of weight sharing using a ResNet-50 model for classifying COVID-19 and achieved a 90.9% sensitivity and 96.9% specificity. An AI-based system was built by Jin et al. [59] to detect COVID-19, with an overall sensitivity of 94.1% and a 95.5% specificity. Xu et al. [58] and Wang et al. [9] present remarkable research and obtained an accuracy of 86.7% and 82.9%, respectively, although just a few performance indicators are mentioned. Our proposed model reported more performance indicators with a satisfactory performance by a margin of 11.96% in accuracy, 2.6% in sensitivity and 4.36% in specificity To detect COVID-19 from CT images, Song et al. [56] adopted a deep learning approach and obtained an 86.0% accuracy. Zheng et al. [47] proposed an 18-layer residual CNN pre-trained on ImageNet with a separate anomaly detection mechanism for the classification COVID-19. The authors recorded an impressive result of 90.7% sensitivity and 90.7% specificity, whereas our model achieved much higher results in comparison to Zhang et al. [41], as depicted in Table 4, with a margin of 9.30% and 9.16% in sensitivity and specificity, respectively. Table 4 summarizes the results of the aforementioned approaches. As seen in Table 4, our proposed model for COVID-19 diagnosis has a competitive efficacy and is capable of handling small-scale dataset with a substantially lower computing cost than well-known deeper neural networks.

According to reference [61], manually detecting COVID-19 with CXR by expert radiologists can have a high sensitivity but a low specificity of 25%. This low specificity leads to false positive predictions, resulting in inefficient therapy and a waste of money. Our proposed model has a high specificity of 99.1% and can be utilized to assist professional radiologists in reducing the number of false positive cases reported.

In addition, some remarks on the computational cost of NW-CapsNet and the model’s complexity are required. We utilized a discrete wavelet multi-resolution analysis, which lowered the model complexity and computation time. Another fascinating characteristic of our NW-CapsNet is its ability to reduce noise in input images by concatenating the sum of the computed detail coefficients at each decomposition level to each convolutional block through a 1×1 convolutional layer. Our model was trained on NVIDIA GTX 1080 in terms of its processing cost. The Keras framework was used to implement the architecture of our proposed model. The complexity of our proposed model is significantly lower than that of previous state-of-the-art models due to the use of fewer training parameters.

## 5. Conclusions

We presented a neurowavelet capsule network for COVID-19 classification in this paper, with the goal of reducing the loss of spatial features by adopting a discrete wavelet multi-resolution analysis to perform down-sampling. For improving the feature extraction robustness of the capsule network, we adopted a multi-resolution analysis of a discrete wavelet transform to filter noise and inconsistent information from the CXR data. Finally, our proposed end-to-end neurowavelet capsule network is used to extract meaningful features from the input CXR images of varying scales for the classification of COVID-19. We have shown that our model has the ability to minimize the loss of spatial details and to learn distinctive details during high-level feature extraction for the classification of COVID-19. By a well-observed margin, our proposed NW-CapsNet performs better than some famous pre-trained models and some previously proposed state-of-the-art COVID-19 diagnosis techniques.

## Figures and Tables

**Table 1:** Description of the chest X-ray dataset showing different categories of pneumonia illnesses and the distribution of images per category, as well as the number of selected images per category.

Pneumonia	Data Count	Selected No	Train Set	Val Set	Test Set
Atelectasis	4999	2980	1788	596	596
Bacteria	3029	2980	1788	596	596
Cardiomegaly	10,000	2980	1788	596	596
Consolidation	10,000	2980	1788	596	596
COVID-19	3616	2980	1788	596	596
Effusion	10,000	2980	1788	596	596
Infiltration	10,000	2980	1788	596	596
Mass	10,000	2980	1788	596	596
Nodule	10,000	2980	1788	596	596
Pneumothorax	10,000	2980	1788	596	596
Healthy	10,000	2980	1788	596	596
Viral	2983	2980	1788	596	596
Total	94,627	35,760	21,456	7152	7152

**Table 2:** Performance analysis between the MRA and max-pooling on NW-CapsNet.

Experiment	ACC (%)	SEN (%)	SPE (%)	PRE (%)	Time (min)
NW-CapsNet (MRA)	99.6	99.2	99.1	99.8	23.2
NW-CapsNet (max-pooling)	95.8	96.4	95.3	96.5	25.3

**Table 3:** Performance comparison of selected deep pre-trained models with the proposed model.

Model	ACC (%)	SEN (%)	SPE (%)	PRE (%)	Time (min)
ResNet-50	97.8	96.7	97.5	98.5	28.1
MobileNet V2	97.4	96.6	97.7	97.8	27.7
Inception V3	97.3	96.2	96.7	96.9	24.2
Inception V2	96.8	95.5	95.1	95.8	26.9
ResNet-101	95.3	93.9	93.7	94.8	27.3
DenseNet	93.8	92.2	92.7	94.2	25.1
NW-CapsNet	99.6	99.2	99.1	99.7	23.2

**Table 4:** Comparison study of the proposed model with state-of-the-art COVID-19 image-based models.

Literature	Architecture	Performance (%)
Wang et al. [9]	2D CNN	82.9 (ACC)
Shi et al. [55]	Random-forest-based CNN	87.9 (ACC), 83.3 (SEN), 90.7 (SPE)
Chen et al. [8]	2D Unet ++	95.2 (ACC), 100.0(SEN), 93.6 (SPE)
Li et al. [45]	2D ResNet 50	90.0 (SEN), 96.0 (SPE)
Song et al. [56]	2D ResNet 50	86.0 (ACC)
Jin et al. [57]	2D Unet++ and 2D CNN	97.4 (SEN), 92.2 (SPE)
Xu et al. [58]	2D CNN	86.7 (ACC)
Jin et al. [59]	2D CNN	94.1 (SEN), 95.5 (SPE)
Wang et al. [31]	3D ResNet and attention	93.3 (ACC), 87.6 (SEN), 95.5 (SPE)
Zhang et al. [41]	2D Unet and 2D CNN	90.7 (SEN), 90.7 (SPE)
NW-CapsNet	Neurowavelet and capsule network	99.79 (ACC), 100 (SEN), 99.86 (SPE), 99.96 (AUC), 100 (PRE)

**Table 5:** Comparison of our proposed NW-CapsNet model with other selected state-of-the-art COVID-19 models using the same training data distribution.

COVID-19 Models	ACC (%)	SEN (%)	SPE (%)	PRE (%)	TIME
COVID-Net [31]	97.9	95.9	96.3	96.9	24.7
DeCoVNet [60]	97.8	96.3	96.4	96.7	26.1
Cov-Net [57]	97.7	95.2	95.8	96.6	23.8
DeepPneumonia [56]	95.1	95.0	96.2	95.6	25.1
NW-CapsNet	99.6	99.2	99.1	99.7	23.2

## Data Availability

The datasets utilized in this work are publicly available in the following links; Link 1: https://www.kaggle.com/tawsifurrahman/covid19-radiography-database (accessed on 5 July 2021). Link 2: https://www.kaggle.com/c/rsna-pneumonia-detection-challenge/data (accessed on 5 July 2021). Link 3: https://www.kaggle.com/nih-chest-xrays/data (accessed on 5 July 2021).
